# Effectiveness, safety, and treatment pattern of sodium zirconium cyclosilicate in Chinese patients with hyperkalemia: interim analysis from a multicenter, prospective, real-world study (Actualize Study)

**DOI:** 10.3389/fphar.2024.1398953

**Published:** 2024-07-29

**Authors:** Nan Shen, Lihong Zhang, Jing Yang, Yongqiang Lin, Xinyu Liu, Xudong Cai, Juan Cao, Qiang Zhu, Xun Luo, Xin Wan, Henglan Wu, Jianming Ye, Chunyan Shan, Hua Xie, Yifan Wu, Yanping Cao, Jianmin Wang, Xiaoyong Yu, Huimin Wang, Jingdong He, Shaojiang Tian, Fenglei Wu, Xinxin Jiang, Lu Li, Li Zuo, Zhaohua Wang, Changying Xing, Xun Yin, Jianrong Zhao, Cong Ma, Gang Long, Qing Li, Yao Hu, Yifan Shi, Hongli Lin

**Affiliations:** ^1^ The First Affiliated Hospital of Dalian Medical University, Dalian, Liaoning, China; ^2^ The First Hospital of Hebei Medical University, Shijiazhuang, Hebei, China; ^3^ Hefei First People’s Hospital, Hefei, Anhui, China; ^4^ Wenzhou Integrated Chinese and Western Medicine Hospital, Wenzhou, Zhejiang, China; ^5^ Nanyang Central Hospital, Nanyang, Henan, China; ^6^ Ningbo Traditional Chinese Medicine Hospital, Ningbo, Zhejiang, China; ^7^ Taixing People’s Hospital, Taizhou, Jiangsu, China; ^8^ Xinghua People’s Hospital, Taizhou, China; ^9^ Hunan Provincial People’s Hospital, Changsha, Hunan, China; ^10^ The First Hospital of Nanjing, Nanjing, Jiangsu, China; ^11^ The First Hospital of Jiaxing, Jiaxing, Zhejiang, China; ^12^ The First People’s Hospital of Kunshan, Suzhou, Jiangsu, China; ^13^ Chu Hsien-I Memorial Hospital of Tianjin Medical University, Tianjin, China; ^14^ Dalian Ruikaer Renal Disease Hospital, Dalian, Liaoning, China; ^15^ Guangdong Provincial Hospital of Traditional Chinese Medicine, Guangzhou, Guangdong, China; ^16^ Handan First Hospital, Handan, Heibei, China; ^17^ Linfen Central Hospital, Linfen, Shanxi, China; ^18^ Shanxi Provincial Hospital of Chinese Medicine, Shanxi, China; ^19^ Liaoning Health Industry Group Bensteel General Hospital, Benxi, Liaoning, China; ^20^ Nuclear Industry 416 Hospital, Chengdu, Sichuan, China; ^21^ Shiyan People’s Hospital, Shiyan, Hubei, China; ^22^ Qidong People’s Hospital Affiliated Qidong Hospital of Nantong University, Jiangsu, China; ^23^ Sandun District of Zhejiang Hospital, Zhejiang, China; ^24^ The First Affiliated Hospital of Xi’an Medical University, Xi’an, Shaanxi, China; ^25^ Peking University People’s Hospital, Beijing, China; ^26^ Taian City Central Hospital, Tai’an, Shandong, China; ^27^ Jiangsu Province Official Hospital, Nanjing, Jiangsu, China; ^28^ Changshu No.2 People’s Hospital, Suzhou, Jiangsu, China; ^29^ The Affiliated Hospital of Inner Mongolia Medical University, Hohho, Inner Mongolia, China; ^30^ Anshan Central Hospital, Anshan, Liaoning, China; ^31^ Tianjin People’s Hospital, Tianjin, China; ^32^ Tianjin Teda Hospital, Tianjin, China; ^33^ Clinical Medical College and Affiliated Hospital of Chengdu University, Chengdu, Sichuan, China; ^34^ AstraZeneca Investment China Co., Medical Affairs, Shanghai, China

**Keywords:** hyperkalemia, sodium zirconium cyclosilicate, safety, real-world clinical practice, Chinese population

## Abstract

**Introduction:** Sodium zirconium cyclosilicate (SZC) is a nonabsorbed cation-exchanger approved in China for the treatment of hyperkalemia [HK; serum potassium (sK^+^) levels >5.0 mmol/L]. This is the first real-world study aimed to assess the effectiveness, safety, and treatment patterns of SZC in Chinese patients with HK. Here we present the results of the first interim analysis.

**Methods:** This multicenter, prospective, cohort study included patients aged ≥18 years with documented HK within 1-year before study enrollment day. These patients were followed up for 6 months from the enrollment day after initiating SZC treatment. The treatment was categorized into correction phase (FAS-P1) and maintenance phase (FAS-P2 new and ongoing users). Subgroup analysis was performed in patients on hemodialysis (FAS-H). The primary objective was evaluation of safety profile of SZC; secondary objectives included assessment of treatment patterns of SZC and its effectiveness.

**Results:** Of 421 screened patients, 193, 354, and 162 patients were enrolled in the FAS-P1, FAS-P2, and FAS-H groups, respectively. sK^+^ levels were reduced significantly from 5.9 mmol/L to 5.0 mmol/L after the correction phase. For the maintenance phase, the mean sK^+^ levels were maintained at 5.2 mmol/L and 5.0 mmol/L in the FAS-P2 new and ongoing user, respectively, and 5.3 mmol/L in the FAS-H subgroup. A considerable proportion of patients showed normokalemia after 48 h of SZC treatment (FAS-P1:51.3%) which was maintained up to 6 months in the maintenance phase (FAS-P2:44%). SZC was well-tolerated.

**Conclusion:** SZC was effective and safe for the treatment of HK in real-world clinical practice in China.

## 1 Introduction

Hyperkalemia (HK), defined as elevated serum potassium (sK^+^) levels beyond the normal range (>5.0 mmol/L), is a potentially life-threatening condition characterized by poor clinical outcomes along with high mortality and morbidity (Amin et al., 2019; Esposito et al., 2020). HK is typically classified by severity levels such as mild (K^+^ 5.0–5.9 mmol/L), moderate (K^+^ 6.0–6.4 mmol/L), and severe (K^+^ ≥ 6.5 mmol/L) (Clase et al., 2020). Severe HK can cause cardiac arrhythmia and arrest, thereby increasing all-cause and cardiovascular mortality if not treated in time (An et al., 2012; McMahon et al., 2012). HK may occur due to the imbalance in K^+^ homeostasis, which is influenced by several parameters such as excessive dietary K^+^ intake, insufficient K^+^ excretion, as well as abnormal distribution of intracellular and extracellular K^+^ (Kovesdy, 2014; Någård et al., 2021). The incidence of HK is even high in patients with comorbidities such as chronic kidney disease (CKD), heart failure (HF), type 2 diabetes, and hypertension, and those treated with renin–angiotensin–aldosterone system inhibitors (RAASis) (Linder et al., 2016). A Chinese epidemiological study found that the incidence rate of HK was 3.86% in the general outpatient population, and patients with CKD, HF, diabetes, and hypertension reported even higher rates of HK (Bian et al., 2021).

HK can be managed through K^+^-restricted diet, administration of dextrose and insulin, intravenous sodium bicarbonate, potassium-binding agents, nonspecific polymeric exchange resins, diuretics, β-agonists, dialysis, and discontinuation or reduced doses of RAASis (Tamargo et al., 2018). Combination treatment using insulin and dextrose is among the most commonly used treatments for the management of HK (Peacock et al., 2018). Insulin reduces sK^+^ levels by activating sodium–potassium adenosine triphosphatase (Allon and Copkney, 1990; Li and Vijayan, 2014). Even though these treatments are rapid, they have a short duration of effect (approximately 4–6 h) and can cause rebound HK. Hence, the combination treatment is rarely considered to be a definitive therapy (Li and Vijayan, 2014). Dialysis is extremely effective in patients with severe HK; however, logistic issues often limit its rapid implementation (An et al., 2012; Peacock et al., 2020). Bicarbonates are only effective in patients with acidosis, and the efficacy of β-agonists can only last for a short period (Rafique et al., 2019). Nonspecific potassium binders, such as sodium or calcium polystyrene sulfonate (SPS/CPS) and patiromer have nonspecific modes of action, higher response time, and may cause severe gastrointestinal (GI) adverse events (AEs) including ischemic colitis, colonic necrosis, and intestinal perforation (Sterns et al., 2010; Chaitman et al., 2016). Therefore, there is a need for additional agents that can safely treat HK in both patients with acute and chronic HK.

Sodium zirconium cyclosilicate (SZC) is an oral, nonabsorbed, potassium binder, which selectively binds to potassium ions and exchanges them for hydrogen and sodium ions throughout the entire GI tract, thereby minimizing the concentration of free potassium in the GI lumen, ultimately lowering the sK^+^ levels and increasing fecal potassium excretion to resolve HK (Stavros et al., 2014). SZC was approved in December 2019 in China for the treatment of HK. Several clinical trials have reported lowering of sK^+^ levels in patients with HK as early as 1 h after the administration of SZC (Kosiborod et al., 2014; Ash et al., 2015; Kosiborod et al., 2015; Packham et al., 2015). The rapid action of SZC is due to its binding capacity to the potassium ions in the upper GI tract, and thus it may serve as a promising alternative treatment to insulin and glucose in the treatment of HK (Amin et al., 2019; Peacock et al., 2020). SZC has shown a promising efficacy and safety profile irrespective of underlying cause of HK, age, sex, race, comorbid diseases, or concomitant use of RAASis (Roger et al., 2019; Ash et al., 2022). Although these clinical trials imply that SZC could be an attractive therapeutic agent, there are very few studies that have evaluated its efficacy and safety in Chinese population in the real-world clinical practice. According to the guidelines from the National Medical Products Administration Drug Intensive Monitoring of Manufacturers, the real-world studies for evaluating the safety and efficacy of SZC are necessary for its extensive use, especially in Chinese population.

Therefore, we conducted this study to evaluate the safety, efficacy, and treatment patterns of SZC in patients with HK. Here, we present the interim results from this study.

## 2 Materials and methods

### 2.1 Study design and participants

The details on study design and methodology has been published previously (Shen et al., 2023). Briefly, this was a multicenter, prospective, noninterventional, cohort study conducted across 40 centers in China in the real-world clinical practice.

Patients aged ≥18 years with sK^+^ levels ≥5.0 mmol/L within 1 year before the study enrollment; currently undergoing SZC, willing to take SZC with physicians’ prescription; and with/without hemodialysis treatment were included. Patients who did not comply with the study protocol guidelines, as well as those who have previously participated in the current study or any other interventional study on the day of enrollment or within the last 3 months, were excluded. Patients were categorized into new SZC users (defined as patients without SZC treatment within 7 days before enrollment and taking SZC on the study enrollment day) and ongoing users (defined as patients with SZC treatment within 7 days before the study enrollment day and continue SZC treatment even after enrollment). All patients were assessed on the 1st, 3rd and 6th months from the study enrolment day and followed up for 6 months. Furthermore, the new SZC user group had an additional follow-up visit on the third day for potassium retesting. The following data were recorded during each visit (from day 1 to month 6, if available): safety and effectiveness data, sK levels, SZC treatment data (if applicable), and any other related data. In addition to the scheduled visits, investigators conducted monthly or additional sK tests as necessary to enhance sK monitoring.

The study was performed according to the Declaration of Helsinki, the International Conference on Harmonization’s Good Clinical Practice, Guidelines for Good Pharmacoepidemiology Practices, and the applicable legislation on noninterventional and/or observational studies. This study received ethical approval from the Ethical Committee of the First Affiliated Hospital of Dalian Medical University (approval number: YJ-JG-YW-2020). All the participating study sites received the ethical approval. Informed consent was obtained from all the included patients before initiating the study.

### 2.2 Treatment regimens

The recommended starting dose of SZC was 10 g, which was administered orally as a suspension in water thrice a day for the first 2 days after enrollment. The patients were switched to maintenance therapy once they reached normokalemia (SZC dosage during maintenance therapy ranged from 5 g on alternate day to 10 g each day). However, these doses were adjusted between 5 g and 15 g once daily for patients on dialysis. The dosage and duration of SZC treatment were decided by the treating physician. Discontinuation of SZC treatment included patients who were not receiving SZC treatment for a period of 7 days or longer. Patients were followed up and documented even after discontinuation from the study.

### 2.3 Study objectives

The primary objective included the evaluation of the safety of SZC in terms of AEs, serious AEs (SAEs), and discontinuation of SZC due to AEs (DAEs). The secondary endpoints included the assessment of AEs, SAEs and DAEs judged by the investigator to be casually related to SZC, the average SZC daily dosage, frequency of different SZC dosages, duration of SZC treatment, dose changes, dose interruption/discontinuation, as well as the sK^+^ levels between the visits and proportion of normokalemic patients with sK^+^ levels between 3.5 and 5.0 mmol/L and 3.5–5.5 mmol/L. This interim analysis evaluated the effectiveness, safety, and treatment patterns of SZC upon enrollment and after follow-up of 1 month after treatment.

### 2.4 Data collection

This study collected patient data from records (such as electronic or paper medical records), local laboratory testing records, and safety data as per investigator’s evaluation in accordance with standard clinical practice. Data were collected for different variables at each visit if applicable. On the enrollment day, data were collected for patient demographic characteristics, medical history, chronic conditions, coronavirus 2019 (COVID-19) vaccination history up to 12 months before enrollment as well as concomitant treatment involving the use of RAASi including angiotensin-converting enzyme inhibitor, angiotensin receptor blocker, mineralocorticoid receptor antagonist, and angiotensin receptor neprilysin inhibitor up to 6 months before enrollment. Parameters such as vital signs, physical examinations, laboratory examinations (renal and heart), biochemical examinations (serum electrolytes, serum creatinine, serum blood urea nitrogen, serum albumin, serum bicarbonate, serum aspartate aminotransferase, and serum alanine aminotransferase) were assessed at each visit up to 6 months after SZC treatment.

### 2.5 Sample size estimations

Considering that 1%–10% of patients with HK have reported DAEs, SAEs, and overall AEs (Roger et al., 2019), the overall sample size of 1,000 patients was planned with 500 patients each in the new user group and the ongoing user group to provide a 95% confidence interval (CI) clinical estimate of 7.4%–12.6% as per the large sample normal approximation method.

### 2.6 Statistical analysis

The full analysis set (FAS), defined as all enrolled patients, was used for this interim analysis. As this study is descriptive in nature, estimates (probabilities, rates, and averages) with the corresponding 95% CIs and supportive descriptive statistics (mean, SD, median, minimum, maximum, and quartiles) were analyzed. The efficacy of SZC treatment was estimated using Kaplan–Meier method. Subgroup analysis was performed as per the SZC dosage time period, that is, administration of at least one dose of SZC during 1–3 days after enrollment in the new user group (FAS-P1), at least one dose of SZC after enrollment in the ongoing user group, and at least one dose of SZC after the completion of the initial period in the new user group (FAS-P2). Additional analyses were performed in patients on hemodialysis (FAS-H) at study enrollment.

## 3 Results

### 3.1 Baseline characteristics

Of the 421 patients comprising the FAS population screened for HK, this interim analysis enrolled 412 patients. There were 193 patients in the first period (FAS-P1) and 354 patients in the second period (FAS-P2). In the FAS-P2 group, 142 (40.1%) patients belong to the new user group, and 212 (59.9%) patients belong to the ongoing user group. The FAS-H group had 162 patients. The mean [standard deviation (SD)] age was 56.9 (14.6), 55.7 (14.4), 58.2 (14.6), and 54.9 (14.1) years in the FAS-P1, FAS-P2 new users, FAS-P2 ongoing users, and FAS-H groups, respectively. Most patients in the intent-to-treat population were males, had stage 5 CKD, and received concomitant calcium channel blockers. No significant changes were observed between the groups with respect to concomitant therapies, including RAASis, diuretics, and antidiabetic drugs. The baseline patient characteristics are presented in Table 1.

**TABLE 1:** Baseline patient characteristics (full analysis set).

Baseline characteristics	FAS-P1 (N = 193)	FAS-P2 new user group (N = 142)	FAS-P2 ongoing user group (N = 212)	FAS-H (N = 162)
Age (years), mean (SD)	56.9 (14.6)	55.7 (14.4)	58.2 (14.6)	54.9 (14.1)
Weight (kg), mean (SD)	66.6 (13.5)	66.2 (13.6)	63.3 (11.1)	62.0 (11.7)
Height (cm), mean (SD)	166.9 (8.3)	166.7 (8.3)	165.3 (7.8)	166.1 (8.3)
BMI (kg/m^2^), mean (SD)	23.7 (3.9)	23.6 (4.0)	23.1 (3.6)	22.3 (3.3)
Gender, n (%)				
Male	121 (62.7)	91 (64.1)	121 (57.1)	94 (58.0)
Female	72 (37.3)	51 (35.9)	91 (42.9)	68 (42.0)
sK^+^ (mmol/L), mean (SD)	5.9 (0.6)	5.2 (0.7)	5.0 (0.6)	5.3 (0.7)
Hemodialysis, n (%)				
Yes	52 (26.9)	37 (26.1)	110 (51.9)	162 (100)
No	141 (73.1)	105 (73.9)	102 (48.1)	0 (0)
CKD staging, n (%)				
Stage 1	0	0	0	0
Stage 2	1 (1.2)	1 (1.9)	3 (3.3)	0
Stage 3	7 (8.2)	6 (11.5)	9 (9.9)	0
Stage 4	14 (16.5)	10 (19.2)	15 (16.5)	0
Stage 5	63 (74.1)	35 (67.3)	64 (70.3)	50 (100)
At least one medical history n (%)	193 (100)	142 (100)	212 (100)	162 (100)
Concomitant medications, n (%)				
Antidiabetics	87 (45.1)	62 (43.7)	75 (35.4)	42 (25.9)
CCB	138 (71.5)	101 (71.1)	157 (74.1)	119 (73.5)
RAAS inhibitors	64 (33.2)	51 (35.9)	85 (40.1)	68 (42.0)
β-blockers	96 (49.7)	74 (52.1)	81 (38.2)	71 (43.8)
Diuretics	61 (31.6)	36 (25.4)	52 (24.5)	15 (9.3)

β-blockers, beta-blockers; BMI, body mass index; CCB, calcium channel blockers; CKD, chronic kidney disease; FAS-P1, patients with at least one dose of SZC, during 1–3 days after enrollment; FAS-P2, patients with at least one dose of SZC, after the completion of the initial period; FAS-H, patients undergoing hemodialysis at study enrollment; N, number of patients with hyperkalemia; RAAS, renin–angiotensin–aldosterone system; SD, standard deviation; s K^+,^ serum potassium; SZC, sodium zirconium cyclosilicate.

### 3.2 Efficacy

Patients with HK were treated with SZC at a mean daily dose of 11.5 ± 10.3 mg/day during the correction phase (FAS-P1 group), 5.0 ± 4.2 mg/day in the FAS-P2 new user group, 6.6 ± 6.5 mg/day in the FAS-P2 ongoing user group, and 6.7 ± 7.5 mg/day in the FAS-H group. The summary of dose changes and their respective reasons for the FAS-P1, FAS-P2 new users, FAS-P2 ongoing user groups, and FAS-H population are given in Table 2.

**TABLE 2:** Summary of dose changes.

	FAS-P1 (N = 193)	FAS-P2 new user group (N = 142)	FAS-P2 ongoing user group (N = 212)	FAS-H (N = 162)
Any dose change	137	89	140	88
Dose change type				
Dose increased	22 (11.4)	20 (14.1)	52 (24.5)	25 (15.4)
Dose reduced	48 (24.9)	47 (33.1)	71 (33.5)	27 (16.7)
Drug interrupted	105 (54.4)	59 (41.5)	101 (47.6)	59 (36.4)
Drug permanently discontinued	7 (3.6)	5 (3.5)	11 (5.2)	11 (6.8)
Reason for dose change				
Adverse event	3 (1.6)	2 (1.4)	18 (8.5)	11 (6.8)
Surgery	0	0	1 (0.5)	1 (0.6)
Patient forgot to take dose	4 (2.1)	4 (2.8)	4 (1.9)	1 (0.6)
Patient’s decision	74 (38.3)	37 (26.1)	51 (24.1)	37 (22.8)
Other	72 (37.3)	60 (42.3)	99 (46.7)	52 (32.1)
Missing	0	0	0	0

FAS-P1, patients with at least one dose of SZC, during 1–3 days after enrollment; FAS-P2, patients with at least one dose of SZC, after the completion of the initial period; FAS-H, patients undergoing hemodialysis at study enrollment; N, number of patients with hyperkalemia; SZC, sodium zirconium cyclosilicate.

**FIGURE 1:**
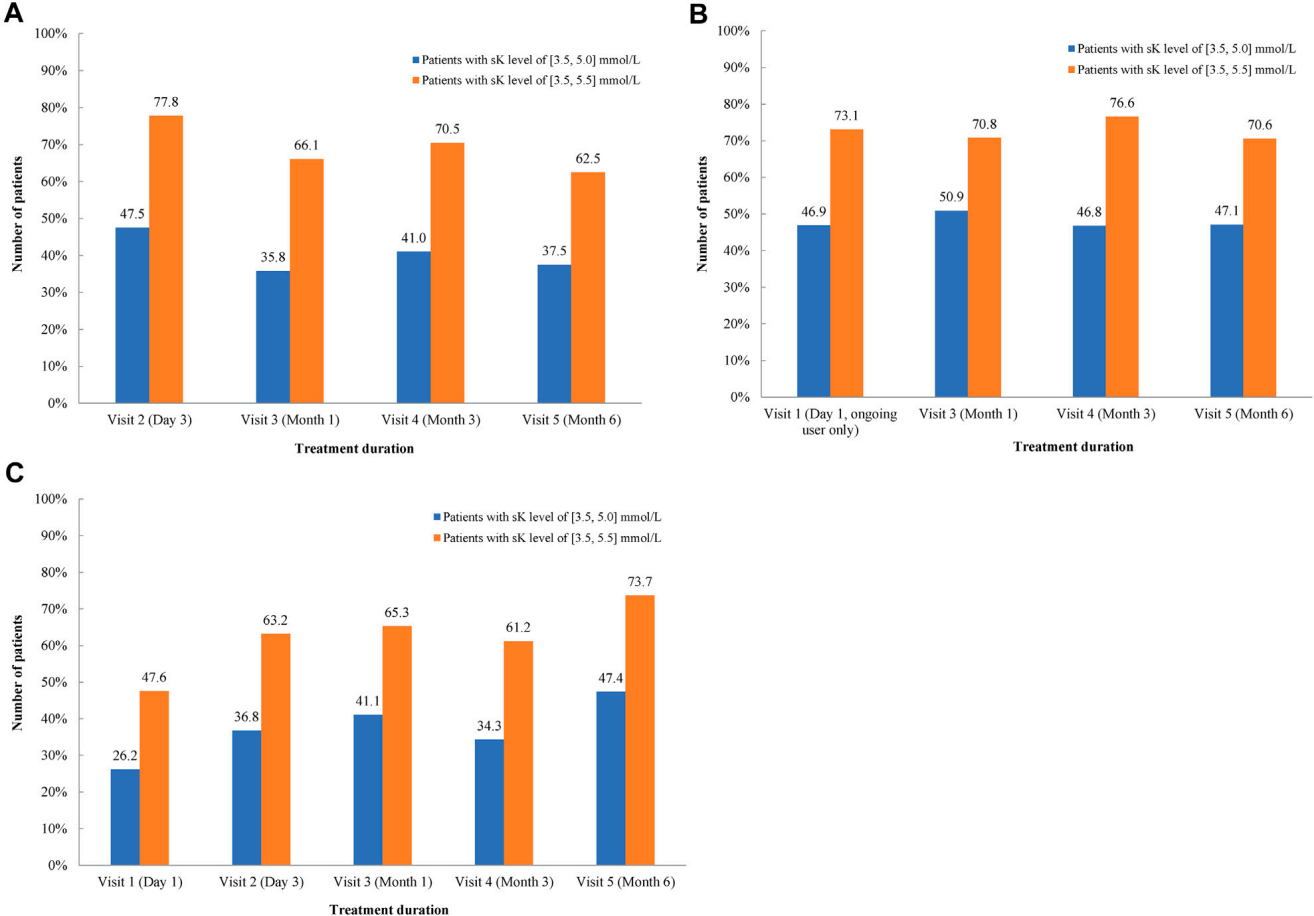
Proportion of patients with the mean serum K^+^ levels between 3.5 and 5.0 mmol/L and 3.5–5.5 mmol/L in FAS-P2 new user **(A)**, ongoing user **(B)** and the FAS-H subgroup **(C)**. FAS-H, patients undergoing hemodialysis at study enrollment; FAS-P2, patients with at least one dose of SZC after the completion of the initial period; N, number of patients with hyperkalemia; SZC, Sodium zirconium cyclosilicate.

In the FAS-P1 group, the mean ± SD sK^+^ levels were reduced from 5.9 ± 0.6 mmol/L at day 1 (visit 1) to 5.0 ± 0.7 mmol/L by day 3, with a difference of −0.9 ± 0.8 mmol/L (95% CI: −1.0, −0.7). The sK^+^ levels of 5.2 ± 0.7 mmol/L, 5.0 ± 0.6 mmol/L, and 5.3 ± 0.7 mmol/L were observed in the FAS-P2 new user group, FAS-P2 ongoing user group, and FAS-H population, respectively, which were lower compared with the sK^+^ levels at baseline. The proportion of normokalemic patients (sK^+^ levels between 3.5 and 5.0 mmol/L) in the FAS-P1 group at visit 2 was 51.3%, whereas the percentage of patients with sK^+^ levels 3.5–5.5 mmol/L at visit 2 was 77.8%. Similarly, the percentage of normokalemic patients (sK^+^ level between 3.5 and 5.0 mmol/L) was 44% in the FAS-P2 group and 47.4% in the FAS-H group at visit 6, whereas the percentage of patients with sK^+^ levels 3.5–5.5 mmol/L was 68% and 73.7% in the FAS-P2 and FAS-H groups, respectively (Figure 1).

### 3.3 Safety

Adverse Events (AEs), Serious Adverse Events (SAEs), and Drug-Associated Events (DAEs) are summarized in Tables 3, 4. Overall, the patients with at least one AE showed an incidence rate per 100 person-days of 1.5 (95% CI: 0.8, 2.8) in the FAS-P1 group, which was higher than 0.4 (0.3, 0.5) and 0.6 (0.5, 0.8) observed in the FAS-P2 new and ongoing user groups, respectively. The FAS-P1 group reported no SAEs, whereas the FAS-P2 new and ongoing user groups reported incidences rates of 0.2 (0.1, 0.3) and 0.3 (0.2, 0.4), respectively. There were no patients with DAEs in all the groups. No patients showed the presence of edema, whereas the incidence of hypokalemia (defined as potassium level < 3.5 mEq/L) was observed to be 0.1 (0.0, 1.0) in the FAS-P1 and FAS-P2 ongoing user groups. Overall AEs for all the groups are summarized in Table 3. The incidence rates of the most common AEs in the FAS-P1 group, FAS-P2 new user group, and FAS-P2 ongoing user group are listed in Table 4, according to the system organ classification. The most commonly occurring AEs include metabolic and nutritional disorders, endocrine disorders, renal and urinary disorders, and GI disorders, which showed the incidence rates per 100 person-days of 0.6 (0.2, 1.5), 0.6 (0.2, 1.5), 0.3 (0.1, 1.1), and 0.3 (0.1, 1.1), respectively in the FAS-P1 population. The FAS-P2 new user group showed common AEs such as infections and infestations, cardiac disorders, and metabolic and nutritional disorders, with the incidence rates per 100 person-days 0.2 (0.2, 0.3), 0.1 (0.0, 0.1), and 0.1 (0.0, 0.1) respectively, whereas common AEs such as infections and infestations, metabolic and nutritional disorders, and GI disorders with incidence rates per 100 person-days 0.3 (0.2, 0.4), 0.2 (0.2, 0.3), and 0.2 (0.1, 0.2), respectively, were observed in the FAS-P2 ongoing user group.

**TABLE 3:** Summary of overall AEs.

Categories	Incidence rate per 100 person days (95% CI)		
	FAS-P1 (N = 193)	FAS-P2 new user group (N = 142)	FAS-P2 ongoing user group (N = 212)
Any AE	1.5 (0.8, 2.8)	0.4 (0.3, 0.5)	0.6 (0.5, 0.8)
Any SAE	0	0.2 (0.1, 0.3)	0.3 (0.2, 0.4)
Any AE leading to drug permanently discontinued	0	0.0 (0.0, 0.1)	0.0 (0.0, 0.1)
Any specific AE			
Edema	0	0	0.0 (0.0, 0.1)
Hypokalemia	0.1 (0.0, 1.0)	0.0 (0.0, 0.1)	0.1 (0.0, 0.1)

AE, adverse event; CI, confidence interval; FAS-P1, patients with at least one dose of SZC, during 1–3 days after enrollment; FAS-P2, patients with at least one dose of SZC, after the completion of the initial period; N, number of patients with hyperkalemia; SAE, serious adverse event; SZC, sodium zirconium cyclosilicate.

**TABLE 4:** The incidence rate of AEs in the FAS-P1, FAS-P2 new and ongoing user groups according to system organ classification.

AEs as per system organ class	Incidence rate per 100 person days (95% CI)		
	FAS-P1 (N = 193)	FAS-P2 new user group (N = 142)	FAS-P2 ongoing user group (N = 212)
Patients with at least one AE	1.5 (0.8, 2.8)	0.4 (0.3, 0.5)	0.6 (0.5, 0.8)
Cardiac disorders	0.1 (0.0, 1.0)	0.1 (0.0, 0.1)	0.1 (0.0, 0.1)
Cardiac failure	0.0 (0.0, 0.0)	0.0 (0.0, 0.1)	0.0 (0.0, 0.1)
Angina Unstable	0.0 (0.0, 0.0)	0.0 (0.0, 0.1)	0.0 (0.0, 0.0)
Ventricular tachycardia	0.1 (0.0, 1.0)	0.0 (0.0, 0.0)	0.0 (0.0, 0.0)
Gastrointestinal disorders	0.3 (0.1, 1.1)	0.0 (0.0, 0.1)	0.2 (0.1, 0.2)
Diarrhoea	0.0 (0.0, 0.0)	0.0 (0.0, 0.0)	0.0 (0.0, 0.0)
Gastrointestinal sounds abnormal	0.0 (0.0, 0.0)	0.0 (0.0, 0.0)	0.0 (0.0, 0.0)
Constipation	0.0 (0.0, 0.0)	0.0 (0.0, 0.1)	0.1 (0.0, 0.1)
Dyspepsia	0.0 (0.0, 0.0)	0.0 (0.0, 0.1)	0.0 (0.0, 0.1)
Vomiting	0.0 (0.0, 0.0)	0.0 (0.0, 0.1)	0.0 (0.0, 0.0)
Psychiatric disorders	0.0 (0.0, 0.0)	0.0 (0.0, 0.1)	0.1 (0.0, 0.1)
Sleep disorder	0.0 (0.0, 0.0)	0.0 (0.0, 0.1)	0.0 (0.0, 0.1)
Anxiety	0.0 (0.0, 0.0)	0.0 (0.0, 0.1)	0.0 (0.0, 0.0)
Insomnia	0.0 (0.0, 0.0)	0.0 (0.0, 0.1)	0.0 (0.0, 0.0)
Musculoskeletal and connective tissue disorders	0.0 (0.0, 0.0)	0.0 (0.0, 0.1)	0.0 (0.0, 0.0)
Chronic kidney disease-mineral and bone disorder	0.0 (0.0, 0.0)	0.0 (0.0, 0.1)	0.0 (0.0, 0.0)
Spinal osteoarthritis	0.0 (0.0, 0.0)	0.0 (0.0, 0.1)	0.0 (0.0, 0.0)
Back pain	0.0 (0.0, 0.0)	0.0 (0.0, 0.0)	0.0 (0.0, 0.0)
Nervous system disorders	0.0 (0.0, 0.0)	0.0 (0.0, 0.1)	0.1 (0.0, 0.1)
Dizziness	0.0 (0.0, 0.0)	0.0 (0.0, 0.1)	0.0 (0.0, 0.0)
Neuropathy peripheral	0.0 (0.0, 0.0)	0.0 (0.0, 0.1)	0.0 (0.0, 0.0)
Restless legs syndrome	0.0 (0.0, 0.0)	0.0 (0.0, 0.1)	0.0 (0.0, 0.0)
Uraemic encephalopathy	0.0 (0.0, 0.0)	0.0 (0.0, 0.0)	0.0 (0.0, 0.0)
Infections and infestations	0.1 (0.0, 1.0)	0.2 (0.2, 0.3)	0.3 (0.2, 0.4)
COVID-19	0.0 (0.0, 0.0)	0.2 (0.1, 0.3)	0.0 (0.0, 0.0)
Upper respiratory tract infection	0.0 (0.0, 0.0)	0.0 (0.0, 0.1)	0.0 (0.0, 0.0)
Pneumonia	0.1 (0.0, 1.0)	0.0 (0.0, 0.1)	0.1 (0.0, 0.1)
Urinary tract infection	0.1 (0.0, 1.0)	0.0 (0.0, 0.0)	0.0 (0.0, 0.0)
Metabolism and nutrition disorders	0.6 (0.2, 1.5)	0.1 (0.0, 0.1)	0.2 (0.2, 0.3)
Hypokalemia	0.1 (0.0, 1.0)	0.0 (0.0, 0.0)	0.1 (0.0, 0.1)
Hyperkalaemia	0.1 (0.0, 1.0)	0.0 (0.0, 0.1)	0.1 (0.0, 0.1)
Hypercalcaemia	0.1 (0.0, 1.0)	0.0 (0.0, 0.1)	0.1 (0.0, 0.1)
Hyperuricaemia	0.3 (0.1, 1.1)	0.0 (0.0, 0.0)	0.1 (0.0, 0.1)
Endocrine disorders	0.6 (0.2, 1.5)	0.0 (0.0, 0.1)	0.1 (0.0, 0.1)
Hyperparathyroidism	0.3 (0.1, 1.1)	0.0 (0.0, 0.0)	0.0 (0.0, 0.0)
Hyperparathyroidism secondary	0.1 (0.0, 1.0)	0.0 (0.0, 0.0)	0.0 (0.0, 0.0)
Thyroid mass	0.1 (0.0, 1.0)	0.0 (0.0, 0.1)	0.0 (0.0, 0.0)
Respiratory, thoracic, and mediastinal disorders	0.1 (0.0, 1.0)	0.0 (0.0, 0.1)	0.1 (0.0, 0.1)
Cough	0.0 (0.0, 0.0)	0.0 (0.0, 0.1)	0.0 (0.0, 0.0)
Respiratory failure	0.0 (0.0, 0.0)	0.0 (0.0, 0.1)	0.0 (0.0, 0.0)
Asthma	0.0 (0.0, 0.0)	0.0 (0.0, 0.1)	0.0 (0.0, 0.0)
Pulmonary arterial hypertension	0.1 (0.0, 1.0)	0.0 (0.0, 0.0)	0.0 (0.0, 0.0)
General disorders and administration site conditions	0.1 (0.0, 1.0)	0.0 (0.0, 0.1)	0.1 (0.0, 0.1)
Complication associated with device	0.1 (0.0, 1.0)	0.0 (0.0, 0.0)	0.0 (0.0, 0.0)
Pyrexia	0.0 (0.0, 0.0)	0.0 (0.0, 0.1)	0.0 (0.0, 0.1)
Chest discomfort	0.0 (0.0, 0.0)	0.0 (0.0, 0.1)	0.0 (0.0, 0.0)
Skin and subcutaneous tissue disorders	0.1 (0.0, 1.0)	0.0 (0.0, 0.0)	0.1 (0.0, 0.1)
Pruritus	0.1 (0.0, 1.0)	0.0 (0.0, 0.0)	0.0 (0.0, 0.1)
Investigations	0.1 (0.0, 1.0)	0.0 (0.0, 0.0)	0.0 (0.0, 0.0)
Blood creatinine increased	0.1 (0.0, 1.0)	0.0 (0.0, 0.0)	0.0 (0.0, 0.0)
Blood urea increased	0.1 (0.0, 1.0)	0.0 (0.0, 0.0)	0.0 (0.0, 0.0)
Injury, poisoning, and procedural complications	0.0 (0.0, 0.0)	0.0 (0.0, 0.1)	0.1 (0.0, 0.1)
Extraskeletal ossification	0.0 (0.0, 0.0)	0.0 (0.0, 0.1)	0.0 (0.0, 0.0)
Haemodialysis complication	0.0 (0.0, 0.0)	0.0 (0.0, 0.1)	0.0 (0.0, 0.0)
Reproductive system and breast disorders	0.1 (0.0, 1.0)	0.0 (0.0, 0.1)	0.1 (0.0, 0.1)
Prostatomegaly	0.0 (0.0, 0.0)	0.0 (0.0, 0.1)	0.0 (0.0, 0.0)
Benign prostatic hyperplasia	0.1 (0.0, 1.0)	0.0 (0.0, 0.0)	0.0 (0.0, 0.0)
Uterine prolapse	0.0 (0.0, 0.0)	0.0 (0.0, 0.1)	0.0 (0.0, 0.0)
Renal and urinary disorders	0.3 (0.1, 1.1)	0.0 (0.0, 0.1)	0.1 (0.1, 0.2)
Acquired cystic kidney disease	0.1 (0.0, 1.0)	0.0 (0.0, 0.0)	0.0 (0.0, 0.0)
Renal cyst	0.1 (0.0, 1.0)	0.0 (0.0, 0.0)	0.0 (0.0, 0.0)
End stage renal disease	0.0 (0.0, 0.0)	0.1 (0.0, 0.1)	0.1 (0.0, 0.1)
Azotaemia	0.0 (0.0, 0.0)	0.0 (0.0, 0.1)	0.0 (0.0, 0.0)
Diabetic end stage renal disease	0.0 (0.0, 0.0)	0.0 (0.0, 0.1)	0.0 (0.0, 0.0)
Hepatobiliary disorders	0.0 (0.0, 0.0)	0.0 (0.0, 0.1)	0.0 (0.0, 0.0)
Gallbladder polyp	0.0 (0.0, 0.0)	0.0 (0.0, 0.1)	0.0 (0.0, 0.0)
Eye disorders	0.0 (0.0, 0.0)	0.0 (0.0, 0.1)	0.1 (0.0, 0.1)
Cataract	0.0 (0.0, 0.0)	0.0 (0.0, 0.0)	0.0 (0.0, 0.0)
Ear and labyrinth disorders	0.0 (0.0, 0.0)	0.0 (0.0, 0.1)	0.0 (0.0, 0.0)
Sudden hearing loss	0.0 (0.0, 0.0)	0.0 (0.0, 0.1)	0.0 (0.0, 0.0)
Vertigo	0.0 (0.0, 0.0)	0.0 (0.0, 0.0)	0.0 (0.0, 0.0)
Blood and lymphatic system disorders	0.1 (0.0, 1.0)	0.0 (0.0, 0.1)	0.1 (0.0, 0.1)
Thrombocytopenia	0.0 (0.0, 0.0)	0.0 (0.0, 0.1)	0.0 (0.0, 0.0)
Anaemia	0.1 (0.0, 1.0)	0.0 (0.0, 0.1)	0.0 (0.0, 0.0)
Congenital, familial, and genetic disorders	0.0 (0.0, 0.0)	0.0 (0.0, 0.0)	0.0 (0.0, 0.0)
Vascular disorders	0.0 (0.0, 0.0)	0.0 (0.0, 0.1)	0.1 (0.0, 0.1)
Aortic dissection	0.0 (0.0, 0.0)	0.0 (0.0, 0.0)	0.0 (0.0, 0.0)
Deep vein thrombosis	0.0 (0.0, 0.0)	0.0 (0.0, 0.1)	0.0 (0.0, 0.0)
Hypertension	0.0 (0.0, 0.0)	0.0 (0.0, 0.1)	0.0 (0.0, 0.0)
Neoplasms benign, malignant and unspecified (incl cysts and polyps)	0.1 (0.0, 1.0)	0.0 (0.0, 0.0)	0.1 (0.0, 0.1)
Acute promyelocytic leukaemia	0.0 (0.0, 0.0)	0.0 (0.0, 0.0)	0.0 (0.0, 0.0)
Cervix carcinoma	0.0 (0.0, 0.0)	0.0 (0.0, 0.0)	0.0 (0.0, 0.0)
Rectal neoplasm	0.0 (0.0, 0.0)	0.0 (0.0, 0.0)	0.0 (0.0, 0.0)
Haemangioma of liver	0.1 (0.0, 1.0)	0.0 (0.0, 0.0)	0.0 (0.0, 0.0)

AE, adverse event; CI, confidence interval; FAS-P1, patients with at least one dose of SZC, during 1–3 days after enrollment; FAS-P2, patients with at least one dose of SZC, after the completion of the initial period; N, number of patients with hyperkalemia; SZC, sodium zirconium cyclosilicate.

The incidence of AEs, as judged by the investigators to be causally related to SZC treatment, was 0.1 (0.0, 0.1) in the FAS-P1 and FAS-P2 ongoing user groups, whereas no incidence of AE was reported in the FAS-P2 new user group. The incidence of AEs in the FAS-P2 ongoing user group by the study index SZC treatment episode duration before enrollment, after 3 days, 1 month, and 3 months is listed in Table 5.

**TABLE 5:** The incidence rate of overall adverse events as judged by the investigator to be causally related to SZC by the study index SZC treatment episode duration before enrollment in the FAS P2 ongoing user group.

Time period before enrollment day	Category	Incidence rate (95% CI), per 100 person days		
		3 days	1 month	3 months
1–3 days	Any AE	0	0.1 (0.0, 0.7)	0.0 (0.0, 0.3)
	Any SAE	0	0	0
	Any AE leading to drug permanently discontinued	0	0	0
	Any specific AE			
	Edema	0	0	0
	Hypokalemia	0	0	0
4–28 days	Any AE	0	0	0
	Any SAE	0	0	0
	Any AE leading to drug permanently discontinued	0	0	0
	Any specific AE			
	Edema	0	0	0
	Hypokalemia	0	0	0
29–84 days	Any AE	0	0.1 (0.0, 0.6)	0.1 (0.0, 0.3)
	Any SAE	0	0	0
	Any AE leading to drug permanently discontinued	0	0	0
	Any specific AE			
	Edema	0	0	0
	Hypokalemia	0	0.1 (0.0, 0.6)	0.1 (0.0, 0.3)
>84 days	Any AE	0	0	0.2 (0.0, 0.7)
	Any SAE	0	0	0.1 (0.0, 0.6)
	Any AE leading to drug permanently discontinued	0	0	0
	Any specific AE			
	Edema	0	0	0
	Hypokalemia	0	0	0.1 (0.0, 0.6)

AE, adverse event; CI, confidence interval; FAS-P2, patients with at least one dose of SZC, after the completion of the initial period; N, number of patients with hyperkalemia; SAE, serious adverse event; SZC, sodium zirconium cyclosilicate.

## 4 Discussion

This interim analysis evaluated the efficacy and safety profiles of SZC after 1 month of treatment in patients with HK (Einhorn et al., 2009; Goyal et al., 2012; McMahon et al., 2012). It has been demonstrated that there was a rapid reduction of sK^+^ levels to normal levels, which were maintained for up to 1 month in patients with HK. This significant reduction of sK^+^ levels was consistent across all patient populations, that is, FAS-P1, FAS-P2 new, and ongoing user as well as FAS-H subgroups. SZC was effective in maintaining normokalemia in patients with HK by reducing sK^+^ levels with good tolerability profile.

This study showed a mean daily dose of 11.5 g in FAS-P1 during the correction phase. During the maintenance phase, a mean daily dose of 5.0 mg/day, 6.6 mg/day, and 6.7 mg/day was used in the FAS-P2 new user group, FAS-P2 ongoing user group, and FAS-H subgroup analysis. This was in line with the previous randomized controlled studies of SZC in patients with HK, which reported the use of SZC doses of 2.5–10 g three times a day (Kosiborod et al., 2014; Ash et al., 2015; Packham et al., 2015) resulting in a clinically significant reduction of sK^+^ levels in these patients with HK, and dosages of 5–15 g QD resulting in sustained normokalemia during the maintenance phase up to 12 months in different clinical studies (Kosiborod et al., 2014; Packham et al., 2015).

The reduced sK^+^ levels of 5.0 mmol/L were observed on day 3 in FAS-P1, 5.2 mmol/L and 5.0 mmol/L in the FAS-P2 new and ongoing user groups and 5.3 mmol/L in FAS-H population, in comparison with 5.9 mmol/L at baseline. The criteria for treatment discontinuation after SZC treatment in patients with HK were not sK^+^ levels 5.0 mmol/L according to the international guidelines, but the actual laboratory report cutoff value of HK (>5.3 or 5.5 mmol/L) at study sites in China, thus reflecting actual treatment patterns of SZC treatment in the real-world clinical practice. This is the first real-world study in China showing reduced sK^+^ levels after SZC treatment. These results are consistent with the previous studies reporting lowering of sK^+^ levels >5.5 mmol/L in 52% of patients within 4 h after the first SZC dose of 10 g dose (Kosiborod et al., 2015). Another phase I study conducted in healthy adults showed significantly decreased sK^+^ concentrations with 10 g of SZC, which were similar to a study conducted in healthy adults in China (Någård et al., 2021; Cheung et al., 2022). In a phase II ZS002 clinical study, a significant decrease in sK^+^ levels was observed in patients with CKD and HK after SZC treatment at a dosage of 3 and 10 g as compared with placebo. There was a reduction in sK^+^ levels after SZC treatment administered at a dose of 10 g for an additional 3.5 days as compared with the placebo group, thereby emphasizing the effectiveness of SZC in the treatment of HK. Also, no significant difference was observed with respect to serum calcium, magnesium, and sodium levels along with other kidney function parameters in both the groups. Similarly, a significant, dose-dependent decline was observed in the mean sK^+^ levels in patients receiving SZC compared with those receiving placebo within the first 48 h in the ZS003 and HARMONIZE clinical studies (Kosiborod et al., 2014; Packham et al., 2015).

SZC treatment was effective with the majority of patients (51.3%) being normokalemic in the FAS-P1 group with sK^+^ levels between 3.5 and 5.0 mmol/L at visit 2, while 77.8% of patients had sK^+^ levels in the range of 3.5–5.5 mmol/L. Similarly, the proportion of patients with sK^+^ levels between 3.5 and 5.0 mmol/L (normokalemic) and 3.5–5.5 mmol/L was 44% and 68%, respectively, in the FAS-P2 group at visit 6. These results were similar to the HARMONIZE study, which showed a rapid attainment of normokalemia in most patients after 10 g SZC TID treatment for 48 h, with a maintenance phase of 29 days with SZC at 5–15 g QD (Kosiborod et al., 2014). The HARMONIZE trial showed normokalemia in 84% and 98% of patients within 24 and 48 h of treatment initiation, respectively (Kosiborod et al., 2014). Another prospective, open-label, single-arm, phase III ZS-005 clinical trial evaluated the long-term efficacy and safety of SZC in 751 outpatients with HK (K^+^ > 5.1 mmol/L) after 12 months of treatment and demonstrated that SZC restored normokalemia in more than 99% of outpatients within 24–72 h in the correction phase. Normokalemia was maintained for up to 12 months in 87% and 99% of participants achieving serum K^+^ ≤ 5.1 mmol/L and ≤5.5 mmol/L, respectively (Spinowitz et al., 2019). The HARMONIZE extension study showed that normokalemia was maintained for up to 11 months during the ongoing SZC QD treatment in 79% of patients, and the treatment was generally well tolerated (Roger et al., 2019).

Furthermore, our study reported that 47.4% of patients to be normokalemic with sK^+^ levels between 3.5 and 5.0 mmol/L, while 73.7% of patients had sK^+^ levels in the range of 3.5–5.5 mmol/L in the FAS-H population at visit 6. Similar results were observed in the DIALIZE study, where a significantly higher proportion of responders was observed in the SZC group compared with the placebo group: 41.2% (n = 40 of 97) versus 1.0% (n = 1 of 99; OR, 68.8; 95% CI, 10.9 to 2810.9; *P* < 0.001) during the evaluation period, which included patients with a maintained predialytic sK^+^ of 4.0–5.0 mmol/L during three or more of four hemodialysis treatments after the long interdialytic interval and who did not require urgent rescue therapy (Fishbane et al., 2019). The DIALIZE China study that evaluated SZC for the management of HK in Chinese patients undergoing hemodialysis showed a significantly higher proportion of responders in the SZC group (37.3%) compared with the placebo group (10.4%), with an estimated OR of 5.10 (95% CI, 1.9–15.1; *P* < 0.001) (Ni et al., 2023). Another ongoing clinical trial, PRECEDE-K, is the first prospective study evaluating the prevalence, recurrence, and treatment pattern in patients with HK undergoing hemodialysis in China and provided high-quality evidence and meaningful insights for guiding physicians in clinical practice (Ni et al., 2021).

SZC was generally well tolerated in this interim analysis, and no major safety concerns were reported. The incidence of treatment-related AEs was comparable, and no major SAEs were reported in all the groups. Although this study lacks data from a control group, thereby restricting the interpretation of safety data, GI disorders are among the most commonly occurring AEs reported in other short-term placebo-controlled studies (Packham et al., 2015; Spinowitz et al., 2019). In this study, reported AEs were generally mild to moderate in severity and manageable. GI AEs were commonly observed in the FAS-P1 and FAS-P2 ongoing user groups with an incidence rate of 0.3 (0.1, 1.1) and 0.2 (0.1, 0.2), respectively. The incidence rate of GI AEs reported in this study is similar to the previous studies showing GI-related disorders in 2.9% of the total patient population, with constipation being the most frequent GI AE (1.8%) (Amin et al., 2019). The HARMONIZE study showed edema to be the most common AE (Packham and Kosiborod, 2016), whereas diarrhea was commonly reported in the ZS-003 study in both the placebo and SZC treatment groups (Ash et al., 2015). A higher incidence of edema observed in HARMONIZE may be due to significantly higher baseline rates of HF and estimated glomerular filtration rate <60 mL/min/1.7 m^2^ and higher baseline levels of brain natriuretic peptide in patients with HK receiving SZC (Kosiborod et al., 2014). However, the present study showed no incidence of edema in all groups. HK is higher in the FAS-P1 and FAS-P2 ongoing user groups with an incidence rate of 0.1 (0.0, 1.0). These results are very similar to previous studies with numerically higher rate of hypokalemia, especially with higher SZC doses due to its mechanism of action (Hoy, 2018). However, hypokalemic patients could be managed via protocol-directed dose adjustments. Nevertheless, monitoring of HK and appropriate dose optimization are essential when administering SZC at higher doses.

Our study demonstrated effective reduction of sK^+^ levels with manageable AEs. This and previous studies of SZC showed that SZC primarily targets potassium ions in the GI tract, is well tolerated with only minor AEs and no SAEs, and is not associated with a large release and systemic absorption of Na^+^ ions. SZC enables effective binding of potassium ions throughout the GIT, which probably explains the significant decrease in sK^+^ levels within 1 h after the administration of the first 10 g dose in patients with HK. These features may also contribute to the low incidence of GI and systemic AEs (Packham et al., 2015).

This interim analysis has several limitations. As this is a real-world study, the effectiveness and safety after SZC treatment is not comparable to that of non-SZC treatment or placebo group. Moreover, a limited number of patients in a specific treatment option may introduce some extent of bias as it is a single-arm study. Also, as this is an interim analysis, effectiveness and safety data of SZC treatment in the long term are awaited.

## 5 Conclusion

The results from the interim analysis of this real-world study demonstrated that SZC was generally well tolerated with low incidences of SAEs and AEs leading to discontinuation in the Chinese population. It was also effective in reducing sK^+^ levels in patients with HK, with a considerable proportion of patients achieving normokalemia after 48 h of treatment and was further maintained up to 6 months in the maintenance phase. Our study results corroborate findings of prior clinical trials and hence SZC could be considered as a good choice for the treatment of acute and chronic HK in China.

## Data Availability

The original contributions presented in the study are included in the article/Supplementary Material, further inquiries can be directed to the corresponding author.
