# Corrigendum: Effectiveness, safety, and treatment pattern of sodium zirconium cyclosilicate in Chinese patients with hyperkalemia: interim analysis from a multicenter, prospective, real-world study (Actualize Study)

**DOI:** 10.3389/fphar.2024.1495716

**Published:** 2024-10-01

**Authors:** Nan Shen, Lihong Zhang, Jing Yang, Yongqiang Lin, Xinyu Liu, Xudong Cai, Juan Cao, Qiang Zhu, Xun Luo, Xin Wan, Henglan Wu, Jianming Ye, Chunyan Shan, Hua Xie, Yifan Wu, Yanping Cao, Jianmin Wang, Xiaoyong Yu, Huimin Wang, Jingdong He, Shaojiang Tian, Fenglei Wu, Xinxin Jiang, Lu Li, Li Zuo, Zhaohua Wang, Changying Xing, Xun Yin, Jianrong Zhao, Cong Ma, Gang Long, Qing Li, Yao Hu, Yifan Shi, Hong-Li Lin

**Affiliations:** ^1^ The First Affiliated Hospital of Dalian Medical University, Dalian, Liaoning, China; ^2^ The First Hospital of Hebei Medical University, Shijiazhuang, Hebei, China; ^3^ Hefei First People’s Hospital, Huaihe Road, Hefei, Anhui, China; ^4^ Wenzhou Integrated Chinese and Western Medicine Hospital, Wenzhou, Zhejiang, China; ^5^ Nanyang Central Hospital, Nanyang, Henan, China; ^6^ Ningbo traditional Chinese Medicine Hospital, Ningbo, Zhejiang, China; ^7^ Taixing People’s Hospital, Taizhou, Jiangsu, China; ^8^ Xinghua People’s hospital, Taizhou, China; ^9^ Hunan Provincial People’s Hospital, Changsha, Hunan, China; ^10^ The First Hospital of Nanjing, Nanjing, Jiangsu, China; ^11^ The First Hospital of Jiaxing, Jiaxing, Zhejiang, China; ^12^ The First People’s Hospital of Kunshan, Suzhou, Jiangsu, China; ^13^ Chu Hsien-I Memorial Hospital of Tianjin Medical University, Tianjin, China; ^14^ Dalian Ruikaer Renal Disease Hospital, Dalian, Liaoning, China; ^15^ Guangdong Provincial Hospital of Traditional Chinese Medicine, Guangzhou, Guangdong, China; ^16^ Handan First Hospital, Handan, Heibei, China; ^17^ Linfen Central Hospital, Linfen, Shanxi, China; ^18^ Shanxi Provincial Hospital of Chinese Medicine, Shanxi, China; ^19^ Liaoning Health Industry Group Bensteel General Hospital, Benxi, Liaoning, China; ^20^ Nuclear Industry 416 Hospital, Chengdu, Sichuan, China; ^21^ Shiyan People’s Hospital, Shiyan, Hubei, China; ^22^ Qidong People’s Hospital Affiliated Qidong Hospital of Nantong University, Jiangsu, China; ^23^ Sandun District of Zhejiang Hospital, Zhejiang, China; ^24^ The First Affiliated Hospital of Xi’an Medical University, Xi’an, Shaanxi, China; ^25^ Peking University People’s Hospital, Beijing, China; ^26^ Taian City Central Hospital, Tai’an, Shandong, China; ^27^ Jiangsu Province Official Hospital, Nanjing, Jiangsu, China; ^28^ Changshu No. 2 People’s Hospital, Suzhou, Jiangsu, China; ^29^ The Affiliated Hospital of Inner Mongolia Medical University, Hohho, Inner Mongolia, China; ^30^ Anshan Central Hospital, Anshan, Liaoning, China; ^31^ Tianjin People’s Hospital, Tianjin, China; ^32^ Tianjin TEDA Hospital, Tianjin, China; ^33^ Clinical Medical College and Affiliated Hospital of Chengdu University, Chengdu, Sichuan, China; ^34^ AstraZeneca Investment China Co., Medical Affairs, Shanghai, China

**Keywords:** hyperkalemia, sodium zirconium cyclosilicate, safety, real-world clinical practice, Chinese population

In the published article, there was an error in **Affiliation 22**. Instead of “Qidong People’s Hospital, The First Hospital of Tsinghua University, Beijing, China,” it should be “Qidong People’s Hospital Affiliated Qidong Hospital of Nantong University, Jiangsu, China.”

The authors apologize for this error and state that this does not change the scientific conclusions of the article in any way. The original article has been updated.

